# Use of Spironolactone for the Treatment of Heart Failure With Preserved Ejection Fraction: Efficacy and Clinical Implications in Light of Recent Evidence

**DOI:** 10.7759/cureus.85908

**Published:** 2025-06-13

**Authors:** Reeju Maharjan, Damilare M Akintunde, Sai Jahnu Sree Reddy Narla, Diana C Baltodano Garcia, Sai Charan Mekala, Sahana Srinivasan, Tuheen Sankar Nath

**Affiliations:** 1 Internal Medicine, Jamaica Hospital Medical Center, New York, USA; 2 Internal Medicine, California Institute of Behavioral Neurosciences and Psychology, Fairfield, USA; 3 Medicine, Medecins Sans Frontieres, Magburaka, SLE; 4 Community Medicine/Public Health, Ahmadu Bello University, Zaria, NGA; 5 Internal Medicine, Mediciti Institute of Medical Sciences, Hyderabad, IND; 6 Medicine, Ricardo Palma University, Lima, PER; 7 Internal Medicine, The Hillingdon Hospital NHS Foundation Trust, Uxbridge, GBR; 8 General Medicine, Government Medical College, Omandurar Government Estate, Chennai, IND; 9 Surgical Oncology, Tata Medical Centre, Kolkata, IND

**Keywords:** efficacy and clinical implications, heart failure, heart failure with preserved ejection fraction, recent evidence, spironolactone

## Abstract

Heart failure with preserved ejection fraction (HFpEF) is a common condition that affects around half of individuals with heart failure (HF), which is associated with significant morbidity, mortality, and reduced quality of life. Even though there is a high prevalence, no pharmacological treatments have been proven to reduce mortality in HFpEF. Spironolactone has shown promise in improving diastolic function and decreasing ventricular stiffness in HFpEF patients. The drug’s mechanism of action, involving aldosterone blockade, may help reduce fibrosis and inflammation, which play a role in the development of HFpEF. We analyzed articles published over the last 18 years using PubMed and the Medical Subject Headings (MeSH) strategy for this review. Using Preferred Reporting Items for Systematic Reviews and Meta-Analyses (PRISMA) guidelines and other inclusion and exclusion criteria, eighteen review articles were selected to assess the efficacy of spironolactone for HFpEF, focusing on randomized controlled trials, meta-analyses, and observational studies. These 18 selected review articles consist of a review of the effects of spironolactone on HF. One of the clinical trials, the treatment of Preserved Cardiac Function Heart Failure (TOPCAT), showed that spironolactone can reduce the incidence of major clinical events, which include cardiovascular death and rehospitalizations. Due to the variability in treatment responses among different treatment groups, personalized treatment may provide optimal results. Such as in America, patients enrolled based on a prior HF hospitalization had significantly higher rates of cardiovascular mortality, HF hospitalizations, and all-cause mortality compared to those enrolled based on B-type natriuretic peptide (BNP) levels. However, in Russia and Georgia, prior hospitalization was not linked to increased risk of death or the primary outcome, though it was associated with more HF hospitalizations, likely because no adjudicated HF events occurred in the BNP group in that region. Even though spironolactone reduces cardiovascular events and improves specific events, its effectiveness is unclear due to the variability in the treatment response and the absence of long-term data. Adverse effects of spironolactone include hyperkalemia, worsening renal function, gynecomastia, and hypotension. This review underscores the need for personalized treatment strategies due to heterogeneity in trial outcomes and further well-designed studies to establish its definitive role in HFpEF management and address critical knowledge gaps in this challenging condition.

## Introduction and background

Heart failure with preserved ejection fraction (HFpEF) affects approximately half of heart failure (HF) patients, contributing to high morbidity, mortality, and diminished quality of life [1]. It poses a significant challenge in the management of HF. Since HFpEF is a heterogeneous condition, with various risk factors such as worsening of kidney function, hyperkalemia, and personalized treatment approaches, individualized treatment responses may be essential for optimizing the outcome [1]. A decline in kidney function in HFpEF resulted in increased incidence of all-cause mortality, recurrent HF hospitalization, cerebrovascular event, and sudden cardiac death. Spironolactone is a mineralocorticoid receptor antagonist (MRA) that has demonstrated the capacity to lower cardiovascular (CV) death and hospitalization in HFpEF in the subset of patients in the Americas TOPCAT study; however, this was not consistent across all regions or the entire cohort [2,3]. Due to myocardial fibrosis, systemic inflammation, and vascular stiffness, there is diastolic dysfunction in HFpEF, where the heart doesn’t relax and fill in properly [4]. The ESC Heart Failure Long-Term registry provides a valuable resource. It demonstrates that while spironolactone's potential for treating HFpEF is encouraging, nothing is known about its long-term safety and effectiveness. Comprehensive data on hyperkalemia and dose escalation in HFpEF patients is lacking due to a substantial gap in the literature [5]. It doesn’t provide the detailed information regarding the risks associated with hyperkalemia, and in multivariable models assessing outcomes such as CV death, HF hospitalizations, and post-enrollment myocardial infarction (MI), the efficacy of spironolactone in HFpEF over the long term [5]. The effects of spironolactone on subgroups of HFpEF patients, particularly those with a history of MI, are not fully understood, despite large trials such as CHARM Preserved, I-Preserve, and TOPCAT. These studies used spironolactone as one of the 32 baseline covariates in multivariable models assessing outcomes such as CV death, HF hospitalizations, and post-enrollment MI rather than analysis of the primary intervention. These studies have demonstrated varying effects across different patient subgroups, such as those with chronic kidney disease, highlighting the need for further research to define which patients are most likely to benefit from spironolactone [6,7]. Although spironolactone has shown a reduction in cardiac fibrosis biomarkers and improvements in diastolic function, it has not been associated with improvements in exercise capacity, highlighting the critical knowledge gap in the use of aldosterone antagonists, like spironolactone, in HFpEF [8]. To determine the potential of spironolactone to reduce morbidity and mortality in HFpEF, the studies should address the gap that focuses on specific patient subgroups, advanced biomarkers, and long-term clinical endpoints is important. Furthermore, the therapeutic efficacy may be enhanced by combining spironolactone with therapies like SGLT2 inhibitors, angiotensin receptor-neprilysin inhibitors (ARNIs), and beta-blockers, and using personalized strategies based on phenotype, biomarkers, imaging, and geography may help overcome the heterogeneity in treatment response seen in HFpEF and enhance its clinical efficacy. This review article focuses on the gaps by exploring current evidence, recognizing areas for future research, and examining the ability of spironolactone as a part of personalized treatment approaches for HFpEF.

## Review

Methods

**Figure 1:**
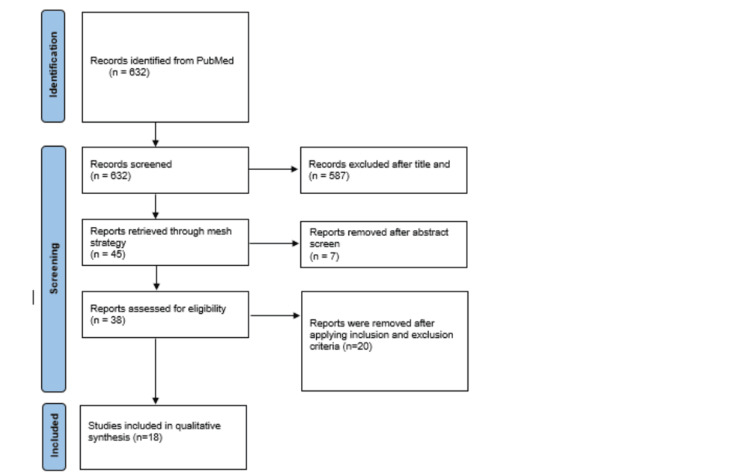
PRISMA Flow Diagram PRISMA: Preferred Reporting Items for Systematic Reviews and Meta-Analyses

The Preferred Reporting Items for Systematic Reviews and Meta-Analyses (PRISMA) guideline of 2009 was used for this systematic review [9]. Different databases, such as PubMed, MEDLINE, and PubMed Central (PMC), were used to retrieve articles. The search strategy included the following keywords: Heart Failure, Spironolactone, Preserved Ejection Fraction, and Efficacy of spironolactone. Medical Subject Headings (MeSH) terms were also implemented to ensure comprehensive coverage of relevant articles (Table 1). A total of 632 studies were retrieved, with regular studies and with a MESH search strategy; 45 review articles were obtained. The titles and abstracts were screened to assess their relevance for inclusion in this review. A total of 41 articles were identified after the initial screening. After careful screening, duplicates were taken out, and the inclusion and exclusion criteria were utilized. Following a detailed analysis, 18 studies were finalized (Figure 1). 

**Table 1:** Keyword Search Results MeSH: Medical Subject Headings

Keywords	Database	Search strategy	Papers identified
( "Spironolactone/adverse effects"[Majr] OR "Spironolactone/agonists"[Majr] OR "Spironolactone/antagonists and inhibitors"[Majr] OR "Spironolactone/chemistry"[Majr] OR "Spironolactone/isolation and purification"[Majr] OR "Spironolactone/metabolism"[Majr] OR "Spironolactone/pharmacokinetics"[Majr] OR "Spironolactone/pharmacology"[Majr] OR "Spironolactone/standards"[Majr] OR "Spironolactone/supply and distribution"[Majr] OR "Spironolactone/therapeutic use"[Majr] OR "Spironolactone/toxicity"[Majr] ) AND ( "Stroke Volume/drug effects"[Majr] OR "Stroke Volume/immunology"[Majr] OR "Stroke Volume/physiology"[Majr] ) AND ( "Heart Failure/diet therapy"[Majr] OR "Heart Failure/history"[Majr] OR "Heart Failure/metabolism"[Majr] OR "Heart Failure/pathology"[Majr] OR "Heart Failure/physiopathology"[Majr] OR "Heart Failure/prevention and control"[Majr] OR "Heart Failure/surgery"[Majr] OR "Heart Failure/therapy"[Majr] )	Pubmed	Mesh (advanced search)	45
Heart Failure, Spironolactone, Preserved Ejection fraction and Efficacy	Pubmed	Regular search	632
Heart Failure, Spironolactone, Preserved Ejection fraction and Efficacy	Google scholar	Regular search	37500
Heart failure	PubMed	Regular search	342071
Spironolactone	PubMed	Regular search	10,300
Heart failure and spironolactone	PubMed	Regular search	2342
Efficacy and spironolactone	PubMed	Regular search	859

Inclusion and Exclusion Criteria

We emphasized studies that included the use of spironolactone in patients with HFpEF. We selected only peer-reviewed articles published in English and articles published in 2013 and onward to include more contemporary research articles. This review article included observational studies, randomized controlled trials (RCTs), and meta-analyses studied in adult patients with HFpEF who were treated with spironolactone. The quality of each study was assessed using the Cochrane Risk of Bias Tool for RCTs, the Newcastle-Ottawa Scale for observational studies, and AMSTAR (Assessing the methodological quality of systematic reviews) for meta-analysis. The studies, which involved patients with heart failure with reduced ejection fraction (HFrEF), pediatric populations, non-English articles, grey literature, case reports, comments, and animal studies, were excluded.

Results

This review included 18 (Table 2) studies with a total of 41,231 HFpEF patients, comprising nine RCTs, eight observational studies (three retrospective and five prospective cohort studies), and one systematic review. One RCT [10] found no significant improvement in peak VO2 or E/e' with spironolactone.

**Table 2:** Summary of Review Articles RCT: Randomized controlled trial, HFpEF: Heart failure preserved ejection fraction, HHF: Hospitalization for heart failure, SZC: Sodium zirconium cyclosilicate, HFrEF: Heart failure reduced ejection fraction, LS: Longitudinal systolic strain, TOPCAT: Treatment of Preserved Cardiac Function Heart Failure with an Aldosterone Agonist

Author and year of publication	Intervention studied	Number of patients	Type of study	Result	Conclusion
Edelmann et al. 2015 [10]	Spironolactone on exercise capacity (peak VO2) and diastolic function (E/e')	422	RCT	After adjusting for various demographic and clinical factors, peak VO2 was not found to have a significant relationship with E/e'.	Demographic and clinical factors interact in varying ways with exercise capacity, resting left ventricular filling index, neurohumoral activation, and the remodeling of the left atrium and ventricle in HFpEF.
Elkholey 2021 [11]	Effects of spironolactone on symptomatic HFpEF.	3445	RCT	A linear relationship was observed between BMI and the impact of spironolactone versus placebo on the primary outcome and cardiovascular death, with the benefit reaching statistical significance at BMI values of 33 kg/m² and 30 kg/m².	In obese patients with HFpEF, the use of spironolactone was linked to a reduced risk of the primary endpoint, cardiovascular death, and HF hospitalizations, when compared to placebo.
Enzan et al. 2020 [12]	The use of spironolactone at discharge	2675	Observational study (retrospective cohort study)	The incidence rate of the primary outcome was lower in patients treated with spironolactone compared to those not receiving it.	Univariate analysis for the combined outcome of all-cause mortality showed that the use of spironolactone at discharge was significantly linked to the primary outcome.
Frankenstein et al. 2013 [13]	The benefit of spironolactone added to ACE inhibitors/angiotensin receptor blockers (ARBs) and beta-blockers	4932	Observational study (retrospective cohort study)	Spironolactone was not associated with improved survival	No association was found between the use of spironolactone and improved survival when given alongside a combination of ACE inhibitors/ARBs and beta-blockers.
Grodin et al. 2018 [14]	Relationship between serum chloride concentration and outcomes in HFpEF	942	Observational study (prospective cohort study)	Reduced serum chloride levels were independently linked to a higher risk of cardiovascular and all-cause mortality in HFpEF.	The use of loop diuretics, but not spironolactone, resulted in a decrease in serum chloride levels over time.
Serenelli et al. 2020 [15]	Mineralocorticoid receptor antagonists (MRAs) on systolic blood pressure (SBP)	4396	RCT	Hypotension was rare and occurred at similar rates with MRA therapy and placebo.	Hypotension was rarely caused by MRA treatment, even when the baseline SBP was low. Therefore, a low SBP should not prevent the use of MRA therapy in patients with HFrEF.
Jhund et al. 2024 [16]	Effect of MRAs in heart failure across the range of ejection fraction	13846	Meta-analysis	In four trials, spironolactone significantly reduced the composite risk of cardiovascular death or heart failure hospitalization overall (HR 0.77; 95% CI 0.72-0.83 0. The benefit was greater in HFrEF patients (HR 0.66; 95% CI 0.59-0.73) than in those with HFmrEF (HR 0.87; 95% Cl 0.9-0.95, *p* for interaction = 0.0012). This article also reviews that comorbidities like diabetes (22% to 41%), hypertension (24% to 91%), and atrial fibrillation (11% to 55%) are highly prevalent in HFpEF patients. Spironolactone efficacy by different ways, such as improving filling pressure and LV function, managing volume overload and fibrosis, and it has anti-inflammatory and anti-fibrotic effects.	The impact of MRA therapy was similar across all subgroups in the trials for HFrEF, HFmrEF, and HFpEF. MRAs provide substantial benefit in HFrEF, significantly reducing CV death, HF hospitalization, and all-cause mortality. In HFmrEF/HFpEF, MRAs mainly reduce HF hospitalizations, with no clear mortality benefit.
Pfeffer et al. 2015 [17]	To understand the difference in clinical outcomes, demographic characteristics of the populations, and their responses to spironolactone	1767	RCT	There was a notable difference in the primary outcome event rates (cardiovascular death, aborted cardiac arrest, or hospitalization for heart failure) between patients from the Americas and those from Russia/Georgia.	Differences in patient characteristics and treatment responses across regions may impact clinical management and outcomes in HFpEF patients.
Kosiborod et al. 2024 [18]	Assess whether sodium zirconium cyclosilicate (SZC) enables MRA use in patients with HFrEF and prevalent hyperkalemia (or at high risk).	365	RCT	The goal is to assess whether SZC allows patients with HFrEF and a risk of hyperkalemia to continue spironolactone, potentially enhancing treatment adherence and outcomes. Hyperkalemia is a shared barrier to spironolactone use in HFpEF. Since SZC effectively controls potassium and spironolactone benefits HFpEF, extrapolating SZC use in HFpEF is reasonable, especially in those with borderline hyperkalemia or CKD	The findings will guide the clinical management of hyperkalemia in heart failure, especially in optimizing MRA therapy. SZC has been shown to enable safe MRA use in HFrEF by maintaining normokalemia, and since spironolactone has shown benefits in reducing HF hospitalization in HFpEF, SZC may play a valuable role in ensuring effective and sustained MRA therapy in this population as well.
Szabo et al. 2024 [19]	Assess history of and time since previous Hospitalization for heart failure (HHF), associations with composite of cardiovascular (CV) death and total HHF, first HHF, and interactions with randomization to spironolactone, in HFpEF	1765	Observational study (Prospective study)	Spironolactone showed a significant interaction with prior HHF. Patients without a history of HHF experienced a greater effect of spironolactone on the composite outcome compared to those with a history of HHF.	Spironolactone was linked to improved outcomes in patients without a history of HHF.
Lund et al. 2024 [20]	To establish the benefit of spironolactone in HFpEF	2400	RCT	It may prove effective in HFpEF and/or HFmrEF, although this has not been definitively established.	It has the potential to enhance the care of patients with HFpEF and/or HFmrEF and revolutionize the approach to clinical trials in heart failure.
McDiarmid et al. 2020 [21]	The myocardial tissue effects of spironolactone in HFpEF	55	RCT	Spironolactone reduced the rate of extracellular expansion, leading to a decrease in the mass of both the cellular and extracellular myocardial compartments.	The mechanism of action of spironolactone in HFpEF involves a direct effect on tissue, including a reduction in the rate of myocardial fibrosis.
Kresoja et al. 2023 [22]	Machine learning approach to identify responders and non-responders to spironolactone among patients with HFpEF	422	RCT	Among the 422 patients in the derivation cohort, iterative cluster allocation identified 159 patients (38%) as responders to spironolactone, in whom E/e' showed significant improvement (p = 0.005).	Machine learning techniques could help identify HFpEF patients who are most likely to respond positively to spironolactone treatment.
Schnelle et al. 2021 [23]	Identify plasma proteins with pathophysiological relevance in HFpEF and with respect to spironolactone-induced effects	386	Observational study (Prospective study)	Plasma biomarkers that can predict spironolactone's impact on all-cause hospitalizations in HFpEF patients.	It offers additional evidence of the direct, beneficial relationship between plasma BNP levels and E/e’ in HFpEF.
Kurgansky et al. 2024 [24]	Effectiveness of spironolactone in reducing death and hospitalization outcomes	3690	Observational study (Retrospective cohort study)	Spironolactone users experienced a 21% reduction (95% CI, 13–29; P<0.0001) in the rate of all-cause death compared to nonusers.	Spironolactone users experienced a 21% reduction (95% CI, 13–29; P<0.0001) in the rate of all-cause death compared to nonusers.
Mentz et al. 2015 [25]	Prognostic utility of left ventricular longitudinal systolic strain (LS) in HFpEF and examines whether LS improves with spironolactone therapy.		Observational study (Prospective study)	In the TOPCAT trial study (n=447), longitudinal systolic strain (LS) <15.8% was linked to a > 2-fold increase in cardiovascular (CV) death, HF hospitalization, or aborted cardiac arrest, independent of EF and diastolic dysfunction.	LS acts as an independent prognostic indicator of poorer outcomes, especially cardiovascular death, in HFpEF patients.
O'Neal et al. 2017 [26]	Relationship between resting heart rate and adverse outcomes in patients with heart failure with preserved ejection fraction (HFpEF).	2705	Observational study(Prospective study)	When the analysis focused on patients not using beta-blockers, the results remained mostly the same, indicating that heart rate is independently linked to worse outcomes, regardless of beta-blocker use.	Lowering heart rate could potentially improve outcomes in these patients, though additional studies are needed to confirm this hypothesis.
Sanders et al. 2018 [27]	Analysis of frailty in relation to outcomes in HFpEF	1767	RCT	Frailty was linked to poorer cardiovascular outcomes and increased mortality.	The effectiveness of spironolactone in improving outcomes in HFpEF was unaffected by the degree of frailty in patients, indicating that frail patients also benefited from the treatment.

Discussion

There are mixed findings across various clinical trials to determine the role of spironolactone in the treatment of HFpEF. It remains a subject of ongoing research. The studies have shown that spironolactone has potential in improving diastolic function and reducing ventricular stiffness in HFpEF patients.

Mechanisms of Action of Spironolactone in HFpEF

Aldosterone is a mineralocorticosteroid receptor agonist. It causes fibrosis, inflammation, and increased myocardial stiffness over a long period of time. All these pathophysiological processes exacerbate diastolic dysfunction in HFpEF. Spironolactone may mitigate these processes by blocking aldosterone, which improves left ventricular compliance and diastolic filling [8,10]. Even though the effects of spironolactone on diastolic function are promising, recent studies have raised questions about its efficacy on fibrotic markers. One of the examples showed galectin-3, a marker of fibrosis, was modestly elevated at baseline in HFpEF patients, and it was continuously rising in the patients despite receiving spironolactone. The use of spironolactone did not significantly change its levels, suggesting that fibrosis in HFpEF might be caused through other pathways that are independent of aldosterone. It showed that even though galectin-3 levels may not be directly associated with the modulation of spironolactone, it could still be a significant marker for providing knowledge into disease progression, clinical outcomes, and prognosis [10]. In addition, the effects of spironolactone on clinical outcomes have shown variable responses based on patient characteristics. Spironolactone was notably associated with a reduced risk of CV death and heart failure hospitalizations (hazard ratio (HR) 0.618) in obese individuals with HFpEF. In contrast, the same treatment with spironolactone had no significant effect on non-obese patients (HR 0.946) [11].

Clinical Implications 

There was a lower incidence rate of the primary composite outcome such as all-cause death or HF rehospitalization compared to the patient population who did not receive spironolactone (30.4% vs. 41.1%), which was demonstrated in the study. This suggests a significant benefit in terms of survival and hospitalization rates [12,28]. The adjusted HR for the primary outcome was 0.63 (95% CI 0.44-0.90; P = 0.010), indicating that spironolactone independently improved prognosis in HFpEF patients, even after accounting for other risk factors such as age, kidney disease, and prior HF admissions [12]. The decision to use spironolactone should be considered after a careful assessment of the individual patient's condition during clinical practice. Despite symptomatic patients being on optimal medical therapy (ACE inhibitors/ARBs and beta-blockers), it may still help manage symptoms by reducing fluid retention, even though it does not significantly impact survival [13]. In addition, the recent studies suggested that serum chloride levels may serve as an important prognostic marker in HFpEF, independent of other clinical variables like natriuretic peptides and sodium levels. While spironolactone does not appear to significantly affect chloride levels or modify the chloride-risk relationship, it remains a valuable treatment for HFpEF by managing fluid retention and addressing the RAAS system [14].

Safety Profile and Tolerability

The study demonstrated that the use of spironolactone in HFpEF has significant benefits. However, its use poses concerns particularly regarding worsening of kidney function and electrolyte balance. The development of worsening renal function (WRF) is one of the significant risks with the use of long-term spironolactone therapy [2,29]. There were significant HRs for adverse outcomes related to renal function, including a HR of 3.1 for CV death, 2.2 for HF hospitalization, and 2.9 for all-cause mortality in patients experiencing WRF [2,29]. The incidence of serious hyperkalemia (potassium >6.0 mmol/L) remains low. However, it occurs more frequently in patients on spironolactone (2.9%) compared to placebo (1.4%) [15]. The ability of spironolactone to mitigate hypokalemia should be noted because the risk of hypokalemia is reduced by 50% with MRAs, with only 7% of patients on spironolactone experiencing hypokalemia compared to 14% in the placebo group [15]. Because spironolactone has a high affinity for the mineralocorticoid receptor but is not specific for it, it also binds to progesterone and androgen receptors, which explains some of its well-known side effects, including gynecomastia and breast pain, erectile dysfunction in men, and irregular menstruation in women who are not yet menopausal. In HFmrEF and HFpEF patients, subgroup analyses revealed that spironolactone increased the risk of both hyperkalemia (OR, 2.56; 95% CI, 1.54-4.27; P<.001) and gynecomastia (OR, 7.82; 95% CI, 3.82-16.01; P<.001), with no subgroup differences (hyperkalemia, P =.67, I2 = 0%; gynecomastia, P =.78, I2 = 0%) [28]. 

Spironolactone can lead to elevated serum creatinine and decreased estimated glomerular filtration rate (eGFR). Regular monitoring of serum creatinine is crucial, as was done in the TOPCAT trial, with a particular focus on preventing creatinine levels exceeding 3.0 mg/dL or any significant increase from baseline values (e.g., doubling the baseline creatinine level) [16]. The incidence of serious hyperkalemia was very notable in clinical studies like EPHESUS, with eplerenone-treated patients showing a 5.9% incidence, compared to 3.5% in the placebo group, representing an absolute increase of 2.4% (P = 0.01) [17]. The more recent MRAs, eplerenone and finerenone, have different pharmacologic profiles (e.g., decreased prevalence of antiandrogenic side effects, including gynecomastia and hyperkalemia, and greater selectivity for mineralocorticoid receptors). These medicines, in contrast to spironolactone, have not undergone thorough testing in HFpEF populations, particularly given the regional diversity observed in TOPCAT. The benefits of spironolactone are thus supported by TOPCAT results in well-characterized HFpEF populations (such as those in the Americas) [17]. The side effects such as hyperkalemia, worsening eGFR, and gynecomastia, can limit the use of spironolactone; thus, other MRAs can be used.

Limitations of the Current Meta-Analysis

Several factors could influence the reported outcomes during the open-label phase despite the inclusion of randomized withdrawal as part of the study design. It may particularly occur when safety or efficacy is evident early in the study. The findings could be impacted when safety analyses are based on patients who received at least one dose of the drug, early treatment discontinuation, or dose modifications due to adverse events. For example, the exclusion of the patient with severe adverse reactions, such as hyperkalemia, may result in an underreporting of the true safety profile of the drug [18]. The limitation may arise if the missing data are not random, and it can introduce selection bias. For example, exclusion of participants with missing data on previous hospitalizations for heart failure (HHFs) and the missing data are systematically related to specific outcomes, it may skew the results and affect the generalizability of the findings [19]. The study included people aged 75 years on average, which may not be the full representation of the general population who have other comorbidities like hypertension and atrial fibrillation. The findings might not be applicable to younger populations or those without these comorbidities, since the study focused on older adults [21]. Lastly, the composite primary endpoint, which includes death from CV causes, aborted cardiac arrest, or HHF is clinically relevant, but combining diverse outcomes could mask the effects of spironolactone on specific components of HF (e.g., hospitalization vs. mortality) or treatment benefits for different subgroups [22]. Future studies should separate key endpoints and conduct component-specific analyses in future research. In Russia and Georgia, spironolactone underuse or poor adherence was revealed by post hoc analyses and drug level evaluations in TOPCAT. This reduces the capacity to extrapolate findings across geographical boundaries and jeopardizes internal validity. The patient's side effect profile should be well controlled in order to increase the adherence to the medication [25].

Heterogeneity and Subgroup Analysis

In Aldo-DHF and TOPCAT trials, there was a variability in treatment responses to spironolactone. These studies highlighted the difficulties in interpreting clinical outcomes across different patient populations. The TOPCAT trial has demonstrated reduced morbidity by improving heart function in certain HFpEF patients; however, it failed to demonstrate consistent clinical improvements, particularly in North American patients. This variability may occur due to differences in the disease genetic factors, phenotype, or even regional variations in healthcare practice and patient populations [23]. In the TOPCAT trial, more diverse populations across different geographic regions (e.g., the Americas, Russia, and Georgia) are included, which may show different results due to differences in demographic, genetic, and environmental factors [24,30]. Subgroup analyses in the literature, such as the results of Shah et al. suggest that impaired longitudinal systolic strain (LS) is a strong prognostic indicator in HFpEF, particularly for CV death, but the utility of left ventricular LS may vary depending on patient characteristics and the specific subgroup being studied [25,31]. Regardless of EF and other standard echocardiographic indicators, abnormal LS (absolute value <15.8%) was linked to a >2x increase in unfavorable outcomes (CV death, HF hospitalization, abortive cardiac arrest). Rather than HF hospitalization, the greatest predictive value was found for CV death. For CV death, LS provided a slight increase in value over conventional risk factors (such as EF and LV size), but it was negligible for composite endpoints. In a subset study, spironolactone was linked to a trend toward better LS over 12-18 months, suggesting a potential function in reducing myocardial strain in HFpEF [25]. Previously, the heart rate was identified as a worse prognosis in HF, with higher rates indicating more severe disease or worse outcomes. The variability of heart rate in patients (categorized in quartiles) could significantly influence the relationship between spironolactone treatment and outcomes such as hospitalization and death. Therefore, the heterogeneity of the patient must be considered when evaluating the effects of spironolactone and other interventions in HFpEF [26].

Recommendations for Future Research

Future longitudinal cohort studies should include serial measurement of frailty using the deficit accumulation approach, the Fried Frailty Phenotype, and other relevant metrics (e.g., gait speed, muscle strength) in HFpEF patients across different stages of the disease should be implemented [27]. The study showed benefits of spironolactone in improving diastolic function and reducing myocardial fibrosis but has failed to show consistent improvements in exercise capacity. This suggests that spironolactone may not significantly impact all aspects of HFpEF [32]. Therefore, trials should include intervention in both pharmacological and lifestyle targeting earlier stages of HF (such as Stage A or B) to delay or prevent progression to more advanced stages of the disease. Randomized controlled trials focusing on patients with high-risk characteristics at these early stages could provide valuable insights into effective strategies for slowing disease [33]. The effects of spironolactone on myocardial fibrosis and diastolic function are well known. To optimize its use in HFpEF, understanding its mechanisms underlying these effects would be beneficial [34]. In addition, future studies should involve longer follow-up periods (5-10 years) to assess the long-term impact of spironolactone on HFpEF progression, CV events, and mortality. The trials should investigate how spironolactone affects functional capacity, patients’ quality of life, and symptom burden. The Kansas City Cardiomyopathy Questionnaire, which is a patient-reported outcome measure, would help to capture a more comprehensive picture of the treatment’s effects on daily living and overall well-being [35]. Future research on HFpEF should use integrated multi-omics approaches to uncover its complex mechanisms. Transcriptomics and proteomics can reveal key gene and protein changes linked to fibrosis, inflammation, and stiffness, while epigenomics can identify regulatory factors influenced by environmental stress. Single-cell and spatial transcriptomics will clarify cellular diversity in heart tissue, and immune profiling can define inflammatory roles, especially in metabolic HFpEF [35].

Impact of Spironolactone

The study evaluated the primary composite outcome of all-cause death, non-fatal myocardial infarction, non-fatal stroke, or HHF over a mean follow-up of 2.8 years. Among 1465 patients, event rates were lower in those taking RAS inhibitors (112.8 vs. 175.4 per 1000 person-years). Adjusted HRs demonstrated significant reductions in primary outcome events (HR 0.75, p=0.01) and all-cause death (HR 0.69, p=0.01). Propensity score-matched analysis confirmed these benefits. The findings suggest that RAS inhibitors reduce CV risks in HFpEF patients with CKD [36]. At baseline and six months after treatment, 158 patients with NYHA class I to II HF and LV ejection fraction ≤40% were evaluated by echocardiography, technetium-99m sestamibi single-photon emission computed tomographic radionuclide ventriculography, gated single-photon emission computed tomography, and cardiopulmonary exercise testing. These patients were randomized to receive spironolactone or a placebo. In the spironolactone group, LV ejection fraction increased from 35.2 ± 0.7% to 39.1 ± 3.5% (p <0.001), with a decrease in LV end-diastolic and end-systolic volumes and myocardial muscle and an improvement in LV diastolic volume. Cardiopulmonary exercise testing parameters did not change [37]. Using biomarkers, researchers assessed collagen cross-linking (CITP:MMP-1 ratio) and collagen deposition (PICP) in 381 patients over a year of spironolactone treatment or placebo. Spironolactone improved left ventricular diastolic dysfunction (LVDD) (reduced E:e' ratio) and lowered collagen deposition (PICP) in patients with lower collagen cross-linking, but not in those with high collagen cross-linking. These findings suggest that excessive collagen cross-linking stabilizes collagen type I fibers, making these patients resistant to the benefits of spironolactone on LVDD [38].

## Conclusions

This review article demonstrates the efficacy of spironolactone for HFpEF. The evidence of effectiveness in HFpEF is evolving, with long-term data assessing the overall benefits and risks of spironolactone. Compared to the patient population that did not receive spironolactone, the study showed a lower incidence rate of the primary composite outcome, such as all-cause death or HF rehospitalization. This points to a notable improvement in hospitalization and survival rates. This article provides an up-to-date, integrated evaluation of spironolactone's efficacy, offering insights that are more timely and relevant. The review article highlights potential sources of bias, concerns regarding generalizability, and issues with the statistical interpretation of the data. The studies should incorporate long-term follow-up periods to assess the sustained benefits of spironolactone on both primary and secondary outcomes. Longer follow-up times should be used in future research to evaluate the long-term effects of spironolactone on the progression of HFpEF, CV events, and death. The effects of spironolactone on patients' quality of life, functional ability, and symptom burden should all be examined in the trials. A thorough picture of how the treatment affects day-to-day functioning and well-being can be obtained with the use of the Kansas City Cardiomyopathy Questionnaire, a patient-reported outcome measure. Research should also focus on investigating the efficacy and safety of spironolactone when combined with other pharmacological agents used in HFpEF, such as SGLT2 inhibitors, ACE inhibitors, and ARNIs, and include targeted strategies (multicenter RCTs, stratified analyses, or real-world registry studies). The future of spironolactone use in HFpEF treatment hinges on expanding and refining research to address these gaps, and we can improve the evidence base for spironolactone in HFpEF.
